# An application of competitive reporter monitored amplification (CMA) for rapid detection of single nucleotide polymorphisms (SNPs)

**DOI:** 10.1371/journal.pone.0183561

**Published:** 2017-08-29

**Authors:** Juliane Havlicek, Eric Rivera-Milla, Peter Slickers, Sönke Andres, Silke Feuerriegel, Stefan Niemann, Matthias Merker, Ines Labugger

**Affiliations:** 1 Alere Technologies GmbH, Jena, Germany; 2 National Reference Center for Mycobacteria, Research Center Borstel, Borstel, Germany; 3 Molecular and Experimental Mycobacteriology, Research Center Borstel, Borstel, Germany; 4 German Center for Infection Research, Partner site Hamburg-Lübeck-Borstel, Borstel, Germany; Defense Threat Reduction Agency, UNITED STATES

## Abstract

Single nucleotide polymorphisms (SNPs) are essential parameters in molecular diagnostics and can be used for the early detection and clinical prognosis in various diseases. Available methods for SNP detection are still labor-intensive and require a complex laboratory infrastructure, which are not suitable for the usage in resource-limited settings. Thus, there is an urgent need for a simple, reliable and rapid approach. In this paper we modified the previously developed competitive reporter monitored amplification (CMA) technique for the detection of resistance mediating SNPs in *Mycobacterium tuberculosis* complex (MTBC) strains. As a proof-of-principle for the application of the CMA-based SNP assay in routine molecular tuberculosis diagnostic, we show that the assay recognizes resistance mediating SNPs for rifampicin, isoniazid and ethambutol from either isolated DNA or heat inactivated *M*. *tuberculosis* cell cultures. The CMA-based SNP assay can identify the most prevalent resistance mediating mutations in the genes *rpoB*, *katG*, *embB*, and the promotor region of *inhA* within one hour.

## Introduction

Single nucleotide polymorphisms (SNPs) are the most frequent types of genetic variations. These mutations can have an impact on phenotypic traits and define genetic modifications of an organism. The detection of SNPs is very important in the molecular diagnostic of diseases if functional relevant genes are affected and for pharmacogenetics applications [1, 2]. The determination of SNPs in genes is widely used for early diagnosis, clinical prognosis and prevention of diseases, e.g. cancer. Over 30 mutations in the BRAF gene have been identified which are associated with human cancer [3, 4]. In addition SNP identification plays an important role for the analysis of drug efficiency with regard to its effective dose, e.g. warfarin dose in coagulation disease [5], or to drug resistance, e.g. in case of an infection with *Mycobacterium tuberculosis* complex (MTBC) strains [6].

Molecular methods to detect SNPs include the utilization of DNA sequencing [7, 8], allele-specific probes [9], nick-translation PCR [10], allele-specific PCR [11], allele-specific ligation [12] as well as an adaption of matrix-assisted laser desorption/ionization time-of-flight mass spectrometry (MALDI-TOF MS) [13]. All these techniques enable a sensitive and reliable SNP detection; however for most of them specialized equipment and trained personnel is required. Due to these aspects these methods are limited for usage in resource-limited settings [14]. To overcome this shortage a simple, rapid and reliable platform for SNP detection is urgently needed.

In our laboratory a molecular test platform for the simultaneous detection and quantification of multiple targets have been developed previously, the Alere q analyzer [15]. This molecular platform combines all required components for the amplification, hybridization and detection in one single-use cartridge and performs all steps automatically once the sample has been applied. The device-specific amplification and detection method, the competitive reporter monitored amplification (CMA), is based on the competitive hybridization of fluorescent labeled oligonucleotides onto an array with immobilized target-specific probes. A first application was a test which is intended to be used for early infant diagnosis in newborns infected with the human immunodeficiency virus [16]. In this paper we modified the CMA method by adjusting assay parameters and the evaluation algorithm to enable the detection of single nucleotide polymorphisms. As representative model we tested its suitability for the detection of SNPs which are associated with antibiotic resistances in case of *M*. *tuberculosis* infections. Resistance towards drugs for treatment of a MTBC infection are mainly mediated by individual SNPs in certain hotspot regions or defined codons of the drug target gene or upstream region, or the drug activating enzyme. In particular for three of four first-line anti-TB drugs the gene *rpoB* (codon 506–533) within the rifampicin resistance determining region (RRDR), codon 306 and 406 in the gene *embB* (mediating ethambutol resistance) and codon 315 in the gene *katG*, as well as the promotor region of *inhA* (both mediating resistance to isoniazid) are epidemiologically and clinically relevant [17–19]. The aim of this study was to detect alterations in these four targets with the CMA-based SNP assay in a selected set of clinical MTBC isolates with Sanger-sequencing determined mutation patterns.

## Materials and methods

### Strains and culture material

The MTBC strains (Table 1) have been isolated at the German National Reference Research Center, Borstel, Germany under standard routine conditions between 2001 and 2006. For the inactivated cell culture material the strains were grown on Löwenstein-Jensen media and subsequently inactivated by ultrasound and heating at 99°C for 15 min each.

**Table 1:** Strains and target-specific plasmids.

ID strain	Plasmid	Mutation
10593/01	p_*rpoB*516Tyr	GAC→TAC
1212/01	p_*rpoB*516Val	GAC→GTC
4724/03	p_*rpoB*526Arg	CAC→CGC
3307/03	p_*rpoB*526Asn	CAC→AAC
2822/06	p_*rpoB*526Asp	CAC→GAC
4787/03	p_*rpoB*526Tyr	CAC→TAC
368/01	p_*rpoB*531Leu	TCG→TTG
4641/01	p_*rpoB*531Trp	TCG→TGG
5472/03	p_*rpoB*533Pro	CTG→CCG
12401/03	p_*katG*315Asn	AGC→AAC
3355/02	p_*katG*315Ile	AGC→ATC
8210/03	p_*katG*315Thr1	AGC→ACC
1429/02	p_*katG*315Thr2	AGC→ACA
8085/03	p_*inhA*-8T>A	T→A
3429/03	p_*inhA*-15C>T	C→T
M.tub_306Ile1	p_*embB*306Ile1	ATG→ATA
7606/01	p_*embB*306Ile2	ATG→ATC
2863/01	p_*embB*306Ile3	ATG→ATT
10109/01	p_*embB*306Leu	ATG→CTG
2822/06	p_*embB*306Val	ATG→GTG

### Generation of plasmid library carrying representative SNPs

To generate a plasmid library various amplicons were cloned into commercial available vectors. The used strains were provided by Research Center Borstel. *M*. *tuberculosis* target-specific sequences were amplified from 1 μL purified DNA with 5 μL of 10x buffer E (Genaxxon bioscience, Ulm, Germany), 0.5 μM of each primer (S1 Table), 250 μM of each deoxynucleoside triphosphate (New England Biolabs, Frankfurt am Main, Germany), 1.5 mM magnesium chloride (Sigma-Aldrich, St. Louis, USA), 2.5 U of *taq* polymerase E (Genaxxon bioscience, Ulm, Germany) in a final volume of 50 μL using a Mastercycler gradient (Eppendorf, Hamburg, Germany) for 2 min at 95°C following 30 cycles at 95°C 15 s, 70°C 30 s, 72°C 30 s and a final elongation step for 15 min at 72°C. The high annealing temperature of 70°C was used due to the strongly decreased formation of side products. Amplicons were purified using the QIAquick PCR purification kit (Qiagen, Hilden, Germany) and cloned into vectors according to the instructions of the TOPO TA Cloning Kit (Invitrogen, Carlsbad, USA). Plasmids were isolated using the PureLink Quick Plasmid DNA Miniprep Kit (Invitrogen, Carlsbad, USA). The sequence of the insert was determined by sequencing with target-specific PCR primers (S1 Table). Plasmid DNA was quantified by the Quant-iT PicoGreen dsDNA Kit (Invitrogen, Carlsbad, USA) and stored at -20°C until analysis.

### Mechanical rupture of culture material and isolation of genomic DNA

To isolate the bacterial genomic DNA of the inactivated culture material the cell suspension was filtered through 5 μm filter pore size (Omnifix Braun, Melsungen, Germany) to obtain single cells. Then the bacteria solution was washed two times with TET buffer (10 mM Tris-HCl, 1 mM EDTA·Na_2_, 0.05% Tween 80), centrifuged at 5,000 × *g* for 5 min and finally adjusted to an optical density of 0.01 in TET buffer. 400 μL of the bacteria cells were disrupted applying a bead beating device (VWR, Erlangen, Germany) and 300 mg of <106 μm glass beads (Sigma Aldrich, St. Louis, USA) at 5,000 × *g* for 80 s. The obtained cell lysate was centrifuged at 5,000 × *g* for 10 s. The supernatant was filtered through a 10 μm pore size filter (Mobitec, Eupen, Belgium) and centrifuged at 5,000 × *g* for 1 min. Subsequently the genomic DNA was extracted from 100 μL cell lysate using the QIAamp Blood Mini Kit (Qiagen, Hilden, Germany) according to manufactures instructions. The isolated DNA was quantified using the Quant-iT PicoGreen dsDNA Kit (Invitrogen, Carlsbad, USA). *M*. *tuberculosis* target-specific amplicon were sequenced by GATC Biotech AG (Konstanz, Germany) and analyzed using the GENtle software (http://gentle.magnusmanske.de).

### Primer, array probes and reporter oligonucleotides

Sequence information for all primer, array probes, TaqMan probes and reporter oligonucleotides is given in S1–S3 Tables. The synthesis of all oligonucleotides was performed by Eurogentec, Cologne, Germany. The reporter oligonucleotides were Cy5 labeled at the 5’ and 3’ end and the array probes had a C7-amino linker for immobilization on the solid phase.

### Efficiency of multiplex target amplification using Real-time PCR

The pre-validation of the multiplex target amplification was performed with 0.5 μM of each *M*. *tuberculosis* sequence-specific primer (S1 Table), 0.2 μM of each TaqMan probe (S3 Table), 0.2 mM of deoxynucleoside triphosphates (Thermo Fisher Scientific, Waltham, USA), 12.5 U of BTR hotstart *taq* (Biotech rabbit, Henningsdorf, Germany), 10μl specific PCR buffer WB2.10 (Alere Technologies GmbH, Jena, Germany) and 10^2^ to 10^6^ copies/μL of genomic (g) DNA from *M*. *tuberculosis* H37Rv reference strain (tebu-bio, Offenbach, Germany) in a final volume of 50 μL using the CFX96 Real-Time System (Biorad, Hercules, USA). The conditions of PCR amplification were: 95°C for 2 min following 40 cycles at 95°C for 30 s, 67°C for 30 s and 72°C for 30 s. To obtain a standard calibration curve serial dilutions from H37Rv DNA varying from 10^2^ to 10^6^ copies/μL were measured and the efficiency for multiplex amplification of *M*. *tuberculosis* specific targets *rpoB*, *katG*, *embB* and the promotor region of *inhA* were calculated. Negative controls were also performed.

### CMA-based SNP assay using the Alere^™^ q analyzer

**Fig 1:**
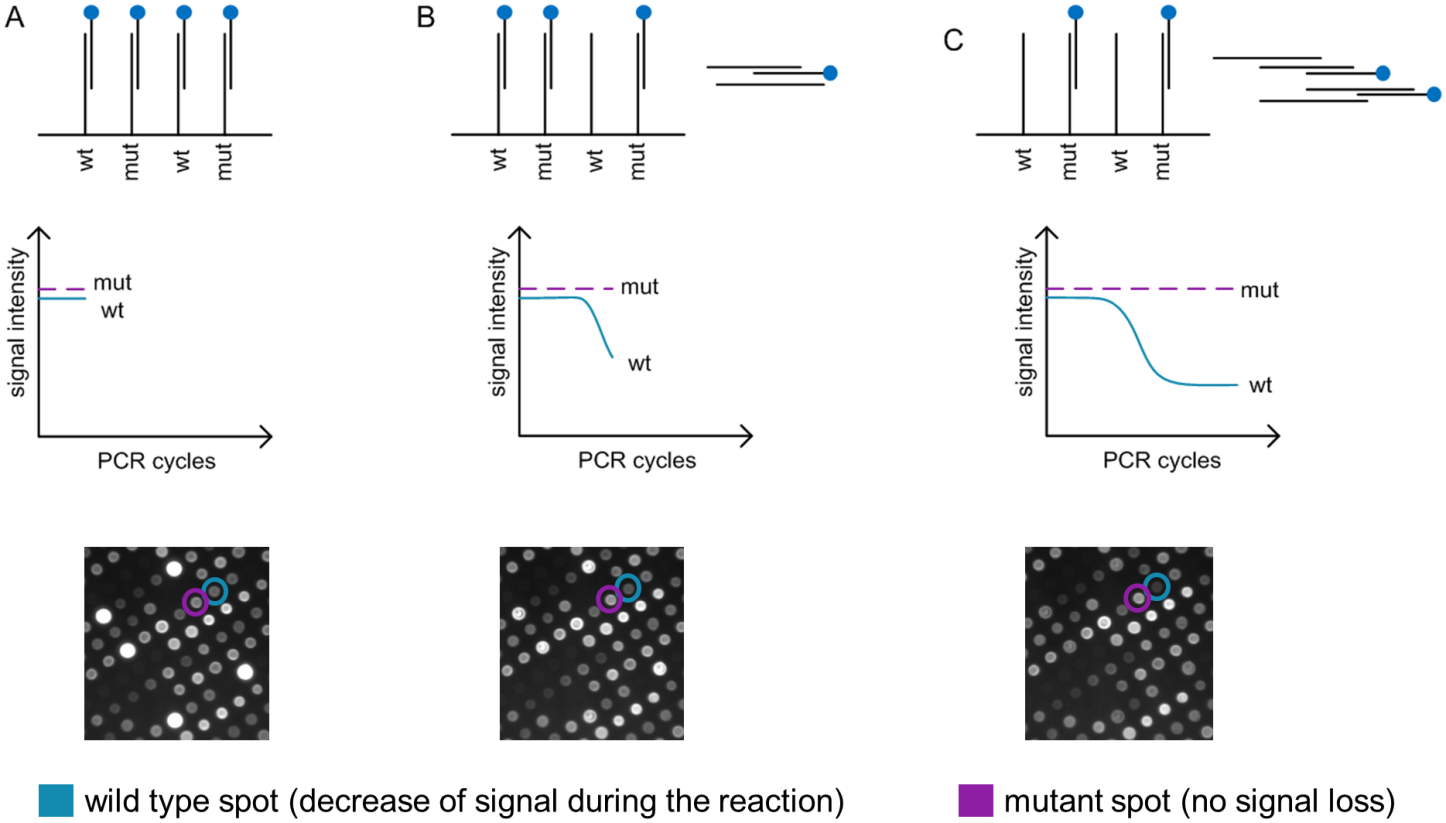
Detection principle of the CMA-based SNP assay. A: For each target there are probes representing the wild type (wt) and mutant (mut) genotypes spotted on a solid phase. Initially the Cy5 labeled reporter oligonucleotides bind to the complementary probes onto the array. B: The template (e.g. wild type DNA) is amplified by PCR and the reporter oligonucleotides bind preferentially to the generated complementary amplicon. A decrease of signal intensity at matched array spots (e.g. wild type probes) is detected. C: The overall signal intensity decreases proportional to the concentration of the amplified target. In this example the signal of the mutant spots remains unchanged.

The description of the Competitive Reporter Monitored Amplification (CMA) and detection principle using the Alere^™^ q assay and Alere^™^ q analyzer was previously published [15]. To apply this technology for detection of *M*. *tuberculosis* specific SNPs, immobilized oligonucleotide probes as well as Cy5 labeled reporter oligonucleotides (S2 Table) carrying the wild type or the corresponding mutation (SNP) in their sequence were used. Initially the reporter oligonucleotides bind to the sequence complementary probes onto the array (Fig 1A). The amplified target compete with the immobilized probes for the binding of the complementary reporter oligonucleotides. Thereby the binding in solution is preferred to the binding on the solid phase (Fig 1B). With progressive reaction more and more reporter oligonucleotides bind to their complementary amplicons which results in loss of fluorescence signal on the corresponding array spots, while on non-corresponding array spots the signal intensity remains (Fig 1C).

The used oligonucleotide array contains different probes for the detection of SNPs within the *M*. *tuberculosis* gene regions *rpoB*, *katG*, *embB* and the promotor region of *inhA* which are associated with drug resistance. Mutations in the *rpoB* gene region are primarily responsible for resistance to rifampicin [17], mutations in the *katG* gene and in the promotor region of *inhA* for resistance to isoniazid [18] and mutations in the *embB* gene for a resistance to ethambutol [19]. With the developed assay different nucleotide exchanges in the respective target regions could be verified (Table 1).

The CMA-based SNP assay was performed in a modified Alere^™^ q cartridge with a final volume of 120 μL comprising 24 μL PCR buffer 5x WB2.10 (Alere Technologies GmbH, Jena, Germany), 0.5 μM of each primer (S1 Table), 4.5 to 5 nM of each reporter (S2 Table), 0.3 mM deoxynucleoside triphosphate mixture (Thermo Fisher Scientific, Waltham, USA), 25 U of BTR hotstart *taq* (Biotech rabbit, Henningsdorf, Germany) and 1 μL plasmid or genomic DNA or inactivated crude cell culture suspension. For negative controls the same reaction mix was used without any template. The amplification was run under the following conditions: 95°C for 2 min following 40 cycles at 95°C for 30 s, 67°C for 30 s and 72°C for 30 s. At the end of each annealing phase an image of the array was acquired. With an annealing temperature of 67°C a sufficient PCR yield and the best discrimination could be achieved.

The melt curve analysis to preselect the best matching reporter variants prior to the assay development was performed without any template in the temperature range from 95°C to 51°C to 95°C in 2°C increments.

### Data processing and analysis

Acquired image series during the amplification were analyzed with the in-house Iconoclust software (Alere Technologies GmbH, Jena, Germany). The array spots were identified using a defined grid and the specific fluorescence signal of each array probe was calculated by subtraction of the non-specific background signal from the absolute value of the triplicate and the averaging of the determined values. To obtain the relative fluorescence of each probe the maximum signal observed during the amplification was considered in relation to the final signal at the end of amplification. The specific genotype was identified calculating the ratio of the relative fluorescence of corresponding pairs of probes representing wild type and mutant sequences, the so-called discrimination factor. A discrimination factor > 1 represented a mutation and a discrimination factor < 1 the wild type. The power to discriminate between wild type and mutations was assessed at different levels. A strong wild type or mutant detection was given if the average discrimination factor -/+ 2SD (standard deviation) was less or more than 1, respectively. If the average discrimination factor -/+ SD was less or more than 1, the reaction was classified as weak wild type or mutant detection. A fifth group was defined if neither wild type nor mutations could be detected.

## Results

### Definition of qualified probe and reporter sets to distinguish SNPs

For the gene regions *rpoB*, *katG*, *embB* and the promotor region of *inhA* in total 27 different variants of probes and reporter oligonucleotides were designed (S2 Table). To select the pairs of reporter oligonucleotides which enable a specific SNP detection and therefore a discrimination between wild type and different mutations, a melt curve analysis was run from 95°C to 51°C to 95°C (in 2°C increments). We determined a specific temperature profile of each reporter oligonucleotide that takes the specific binding efficiencies into account. In case of low fluorescent signals reporter oligonucleotides were excluded from further analysis (S1A Fig), in total this affected nine reporter oligonucleotide variants. As second parameter the binding of a reporter oligonucleotide to its matched and unmatched probes was analyzed and the difference in temperature at a defined signal level (0.005) determined. This calculated value was used to estimate the potential for a reporter oligonucleotide to discriminate between wild type and mutations. If the reporter oligonucleotide binding between the matched and non-matched probe variants showed a clear difference in temperature, the reporter oligonucleotides were selected for further assay development (S1B1 Fig), otherwise were not applied (S1B2 Fig). Using this parameter eight more reporter oligonucleotides were excluded to avoid unspecific probe binding within the assay. Finally all reporter oligonucleotide variants which were preselected by these two parameters were analyzed using plasmid DNAs representing the wild type and the corresponding mutant genotype for all *M*. *tuberculosis* specific target regions in different combinations. The reporter oligonucleotide pairing yielding the most distinct identification of SNPs was defined for the final complete reporter set (selection in S2 Table).

### Identification of SNPs in MTBC specific target regions using plasmid DNA

The efficiency of the multiplex amplification for chosen targets *rpoB*, *katG*, *embB* and the promotor region of *inhA* was found to be > 0.9 (S2 Fig). To analyze the performance of the developed assay to identify specific SNPs, a set of 20 plasmids carrying defined mutations was investigated. In a first experiment series the amplification was performed in the presence of all target-specific primers and reporter oligonucleotides for one of the four target regions. If genomic wild type DNA was applied to the reaction a discrimination factor < 1 was determined on all probes. Contrary in experiments using plasmids carrying mutant genotypes a discrimination factor > 1 was observed on the corresponding probe which indicated that all reactions were highly specific. In a few cases a reaction was also observed on non-corresponding probes; however the SNP could be correctly identified as the value for the discrimination factor of non-corresponding probes was clearly lower compared to the matched genotype (S4 Table). In a next experiment series all target-specific primers and reporter oligonucleotides were added to the reaction. Genomic wild type DNA and selected plasmids with defined mutations (*rpoB*526 CAC→GAC, *rpoB*531 TCG→TTG, *katG*315 AGC→AAC, *katG*315 AGC→ATC, *katG*315 AGC→ACC, *katG*315 AGC→ACA, *inhA*-15 C→T, *embB*306 ATG→ATC and *embB*306 ATG→GTG) were investigated. All probes showed a discrimination factor < 1 if genomic wild type DNA was tested. All plasmids carrying mutations were clearly identified showing the highest discrimination factor on the sequence-matched probe (S5 Table). To simulate the situation of multidrug resistance, plasmids carrying different mutations were analyzed in one experiment. The following combinations were tested: *rpoB*526 CAC→GAC + *embB*306 ATG→GTG, *rpoB*531 TCG→TTG + *katG*315 AGC→ATC, *rpoB*526 CAC→GAC + *katG*315 AGC→ACA + *embB*306 ATG→GTG and *rpoB*531 TCG→TTG + *katG*315 AGC→ATC + *embB*306 ATG→ATC in comparison to genomic wild type DNA. In case of genomic wild type DNA a discrimination factor < 1 was consistently determined on all probes. All analyzed mutations could be correctly identified by a discrimination factor > 1 on corresponding probes regardless of the number of analyzed plasmids. In some cases reactions could be also observed on non-corresponding probes. However, the correct SNP could be still detected by considering the probe with the highest discrimination factor (S6 Table).

### Sensitivity

**Fig 2:**
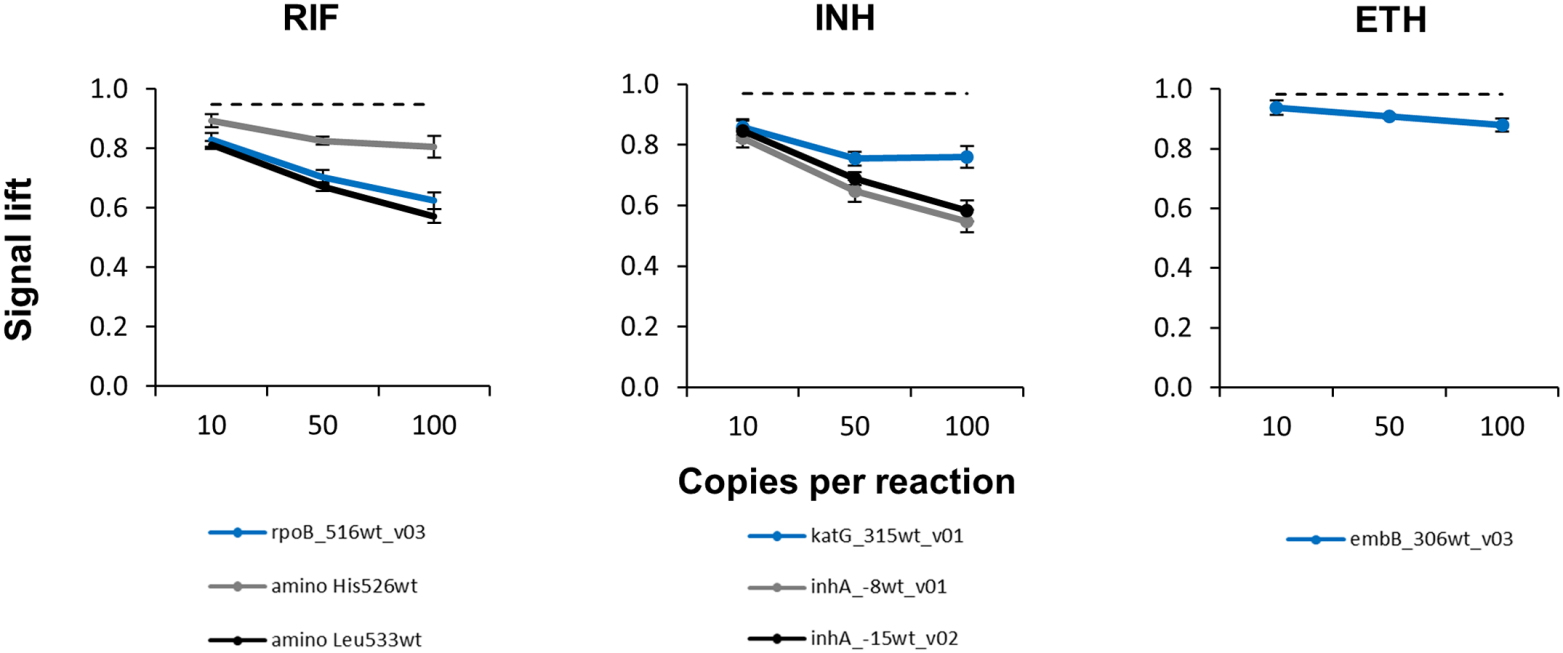
Determination of the CMA-based SNP assay sensitivity. Multiplex amplification was performed with varying DNA concentrations of the *M*. *tuberculosis* reference strain H37Rv. The y-axis represents the average signal lift which is the ratio of the maximum and the final signal at the respective wild type probe. The error bars indicate the standard deviation of the experiment series. A control reaction which did not contain any template DNA is shown as a dashed line.

The sensitivity of the amplification was determined by using purified genomic DNA from *M*. *tuberculosis* reference strain H37Rv at concentrations of 10, 50 and 100 copies per reaction. All targets *rpoB*, *katG*, *embB* and the promotor region of *inhA* were amplified in a multiplex reaction and the signal lift was determined (Fig 2). This signal lift was defined as the ratio of the maximum and final fluorescence signal and it declined according to the template copy number used in the reaction. For the gene targets *rpoB*, *katG*, *embB* and the promotor region of *inhA* the detection was possible down to 50 copies per reaction and could be clearly differentiated from reactions without any template.

### Application of CMA for clinical MTBC isolates

**Fig 3:**
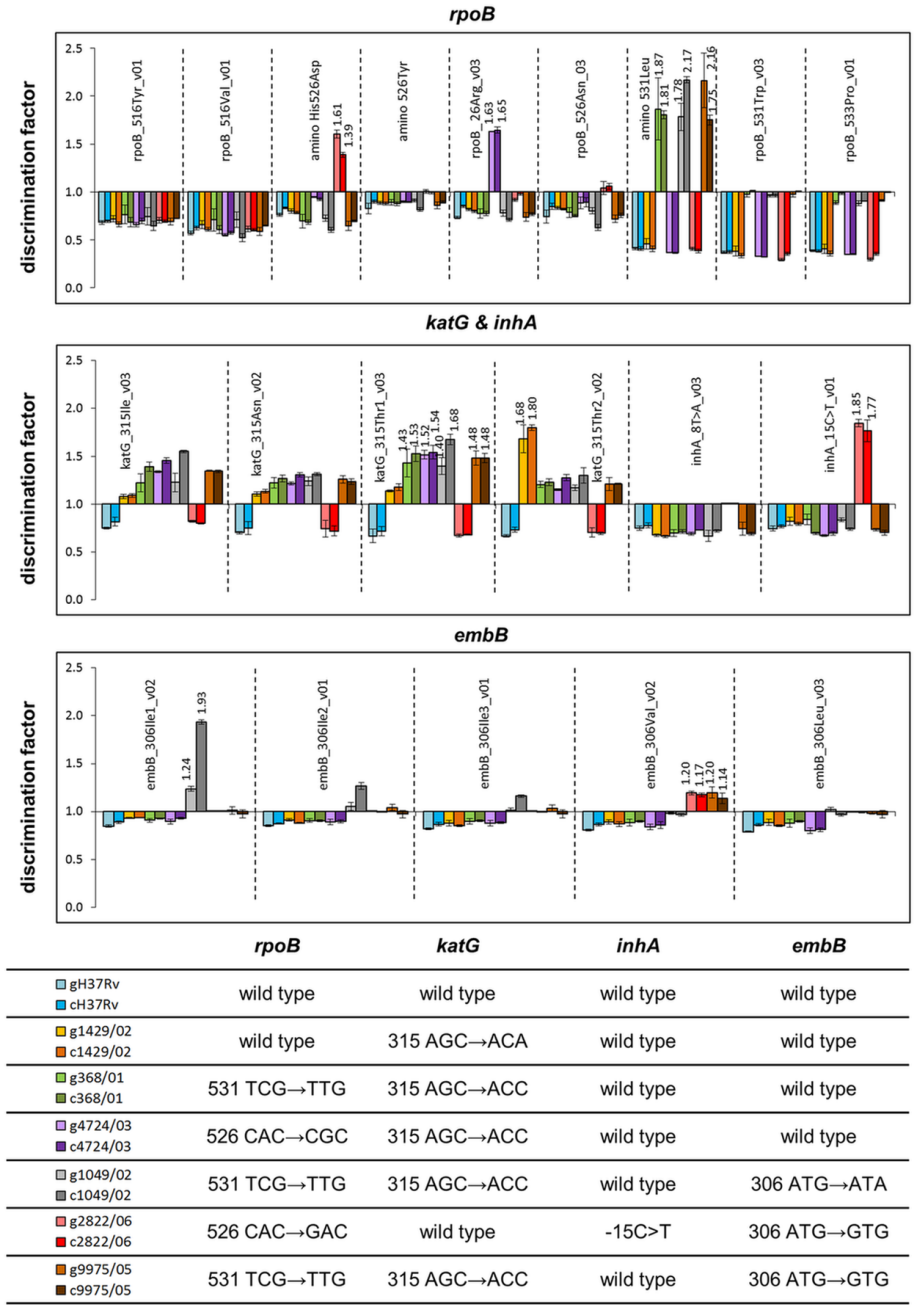
Analysis of genomic DNA (g) and heat-inactivated cell culture material (c) from different *M*. *tuberculosis* strains. The target-specific genotypes (*rpoB*, *katG*, *embB*, promotor region of *inhA*) of analyzed strains are summarized in the table below. The test results are given as discrimination factors (y-axis) and present the average of three measurements. The x-axis shows the different mutant probes in the corresponding target region. The discrimination factors are given for the reaction of a mutation on its corresponding probe. All SNPs of the particular strain isolates could be identified precisely by a discrimination factor >1 which is characteristic for a mutation and by discrimination factor < 1 at probe positions which represented the wild type. No difference in reactivity was observed when inactivated crude culture material was applied compared to genomic DNA. The test results for all strains are available in S4 Table.

To verify the ability of the CMA-based SNP assay to identify SNPs within clinical isolates, a selection of MTBC strains was analyzed. Based on their molecular resistance profiles the isolates were classified in three groups, i.e. mono-resistant isolates, isolates exhibiting two and three resistance mediating mutations, respectively. On all probes a discrimination factor < 1 was observed if genomic wild type DNA was tested (Fig 3). Within group 1 isolates were defined by their monoresistance. As representative the isolate 1429/02 (*katG*315 AGC→ACA) was analyzed and could be correctly identified by the highest discrimination factor on its corresponding probe. The isolates 368/01 and 4724/03 in the defined group 2 carried double mutations in *rpoB*531 TCG→TTG, *katG*315 AGC→ACC and in *rpoB*526 CAC→CGC, *katG*315 AGC→ACC, respectively. These mutations could be clearly identified in both isolates (Fig 3). Within group 3 the isolates carried mutations in three different target regions. The isolate 1049/02 was characterized by the mutations *rpoB*531 TCG→TTG, *katG*315 AGC→ACC and *embB*306 ATG→ATA, the isolate 2822/06 by the mutations *rpoB*526 CAC→GAC, *inhA*-15 C→T and *embB*306 ATG→GTG and the isolate 9975/05 by the mutations *rpoB*531 TCG→TTG, *katG*315 AGC→ACC and *embB*306 ATG→GTG. For all isolates of this group the genotype was determined correctly (Fig 3).

### Assay performance using crude culture material

To characterize the assay performance with crude sample types and thus the potential to be used in a non-laboratory environment, inactivated culture material from TB patient isolates were applied to the assay. Using only 1 μL of the cell extracts we could clearly identify all mutations as well as with the genomic DNA purified from these cell suspensions (Fig 3). Notably, our assay was able not only to detect SNP variants affecting one gene (e.g. sample 1429/02), but also simultaneous identify SNPs on two (e.g. sample 368/01) or three (e.g. 2822/06) genes. The direct usage of inactivated culture material within a cartridge which can be processed fully automatically from the amplification to the detection given a result within one hour could have a great advantage in resource-limited settings.

## Discussion

The CMA-based SNP assay has been shown to be a rapid, easy and reliable method for the detection of single nucleotide polymorphisms in clinical MTBC isolates. All defined SNPs and SNP combinations could be correctly identified using plasmid DNA, genomic DNA and inactivated culture material. Using an extended resistance gene target probe set the CMA-based SNP assay has the potential to be deployed as point of care diagnostic test for molecular drug resistance testing of clinical MTBC isolates.

We adapted our previously published CMA approach on the molecular Alere^™^ q platform [15] for the rapid detection of SNPs. As a proof-of-principle of the CMA-based SNP assay we identified mono- and multidrug-resistant MTBC strains in crude cell lysates (S7 Table) without the need of additional sample preparation. This makes a test system suitable for its application in low level laboratory environments where no cost intensive laboratory equipment is available. Especially in settings where the laboratory infrastructure is limited the usage of crude sample extracts could facilitate drug resistance diagnosis. Compared to the CMA-based SNP assay, standard SNP detection methods, e.g. allele-specific PCR or allele-specific ligation are very laborious and time-consuming as the amplification und detection are processed in two steps with different methods and instruments [11, 12]. In addition the quality of the analyzed template is crucial for the test result [20].

The suitability of SNP genotyping methods for the clinical diagnosis is also depended on their sensitivity and accuracy [21]. The detection of MTBC DNA was possible with a sensitivity down to 50 copies per reaction with the described molecular assay which is comparable to standard SNP detection methods with molecular beacons [22, 23] and TaqMan probes [24, 25]. With the CMA-based SNP assay all genotypes of the sequenced isolates could be correctly identified (Fig 3 and S7 Table) indicating that the test accuracy is as high as for alternative SNP methods.

Further the degree of multiplexing is an essential parameter for the choice of the appropriate SNP method. The CMA-based SNP assay described herein used 27 different probes immobilized on an array for the detection of the major mutations causative for rifampicin, isoniazid and ethambutol drug resistance in MTBC strains. This array technology is of great advantage to Real-time PCR based systems, which are limited by the availability of multi-color detection channels. Prospectively it would be possible to increase the array resolution by a high density spotting technology enabling SNP detection with up to 200 different probes by this molecular platform. It is possible to expand the developed test for the detection of SNPs which are mediating a resistance towards other antituberculosis drugs (e.g. amikacin, bedaquiline or delamanid). In case of a more complex gene analysis DNA sequencing technologies are the method of choice [26] and new genetic markers can be discovered by e.g. next generation sequencing [27, 28].

In conclusion the CMA-based amplification and detection is a fast, sensitive and simple method for the molecular detection of SNPs. It offers the potential to diagnose drug resistance mediated by well characterized SNPs and can be applied on crude cell material with a short turn-around time of one hour which makes this molecular assay attractive for the application in resource-limited settings.

## Supporting information

S1 Fig Exemplary melt curve analysis of selected probe and reporter variants. For preselection of reporter oligonucleotide variants a melt curve analysis was done. A: In a first step only reporters with signal intensities > 0.5 were selected (*rpoB* 531 wild type). Therefore the reporter rpoB_531_v01 was excluded due to its low signal intensity of 0.4. B: As second parameter the binding strength of a reporter to its corresponding and non-corresponding probe variants was determined (*rpoB* 516) by the difference in temperature at a defined signal level (0.005). Based on this value the reporter rpoB_516wt_v03 was selected for further experiments (B1) whereas the reporter amino Asp516wt was excluded (B2) to lower the risk for a weak discrimination between wild type and mutations.
(PDF)

S2 Fig PCR efficiency of the multiplex amplification for all defined *M*. *tuberculosis* specific targets. The multiplex amplification of the targets *rpoB*, *katG*, *embB* and the promotor region of *inhA* was determined using *M*. *tuberculosis* sequence-specific TaqMan probes by Real-Time PCR. Different concentrations of genomic DNA of the *M*. *tuberculosis* reference strain H37Rv were tested. The x-axis shows the DNA concentration and the y-axis represents the determined cycle threshold (n = 8). For each target the calibration curve and the calculated efficiency are given which were found to be excellent (E > 0.9).
(PDF)

S1 Table Primer used for the preparative PCR and for the CMA-based SNP assay. (PDF)

S2 Table Probe and reporter variants used for the development of the CMA-based SNP assay. (PDF)

S3 Table Target-specific TaqMan probes used for the determination of the Real-Time PCR efficiency. (PDF)

S4 Table Analysis of wild type genomic DNA and target-specific plasmids carrying different mutations in a multiplex amplification reaction applying all reporter oligonucleotides within the respective target region. The table shows the determined discrimination factors in the upper and the corresponding standard deviations in the lower row, respectively (n = 3 to 4). A discrimination factor < 1 indicates a wild type and a discrimination factor > 1 indicates a mutation. Additionally there is a differentiation between a weak and a strong wild type and mutant detection, respectively. A strong detection is given if the average discrimination factor -/+ 2SD is clearly < 1 for a wild type and > 1 for a mutant detection. If the average discrimination factor -/+ SD is in the range for a wild type or a mutant it is a weak detection.
(PDF)

S5 Table Analysis of wild type genomic DNA and target-specific plasmids carrying different mutant genotypes in a multiplex amplification reaction applying all reporter oligonucleotides for all defined target regions. The table shows the determined discrimination factors as well as the standard deviations (n = 3 to 5) for all analyzed target-specific plasmids. A strong wild type or mutant detection is given if the average discrimination factor -/+ 2SD is <1 or >1, respectively. A weak detection was determined if the average discrimination factor -/+SD was < 1 or > 1, respectively.
(PDF)

S6 Table Analysis of different plasmid combinations with the CMA-based SNP assay. The table shows the determined discrimination factors and the standard deviations, respectively (n = 3 to 4) if plasmids carrying different mutations were combined. A strong wild type or mutant detection is given if the average discrimination factor -/+ 2SD is < 1 for a wild type and > 1 for a mutant, respectively, whereas values of < 1 or > 1 for the average discrimination factor -/+SD represents a weak detection.
(PDF)

S7 Table Analysis of genomic DNA and inactivated cell culture material from different *M*. *tuberculosis* strains. The results of tested genomic DNAs (g) and inactivated cell culture material (c) derived from the same strain are listed in the table (n = 3 to 4). The discrimination factors are given in the upper and the standard deviations in the row below, respectively. A wild type is detected if the discrimination factor is < 1. A differentiation is applied between a strong (average discrimination factor + 2SD) and a weak (average discrimination factor + SD) wild type detection. A strong mutant detection is given if the average discrimination factor − 2SD is clear > 1 and a weak mutant detection for an average discrimination factor—SD is > 1.
(PDF)
