# Supplementary material for: An application of competitive reporter monitored amplification (CMA) for rapid detection of single nucleotide polymorphisms (SNPs)
Source: PLoS One. 2017 Aug 29;12(8):e0183561. doi: 10.1371/journal.pone.0183561 (PMC5574540; doi:10.1371/journal.pone.0183561)
Supplement: S1 Fig — For preselection of reporter oligonucleotide variants a melt curve analysis was done. A: In a first step only reporters with signal intensities > 0.5 were selected (rpoB 531 wild type). Therefore the reporter rpoB_531_v01 was excluded due to its low signal intensity of 0.4. B: As second parameter the binding strength of a reporter to its corresponding and non-corresponding probe variants was determined (rpoB 516) by the difference in temperature at a defined signal level (0.005). Based on this value the reporter rpoB_516wt_v03 was selected for further experiments (B1) whereas the reporter amino Asp516wt was excluded (B2) to lower the risk for a weak discrimination between wild type and mutations. (PDF) [file pone.0183561.s001.pdf]

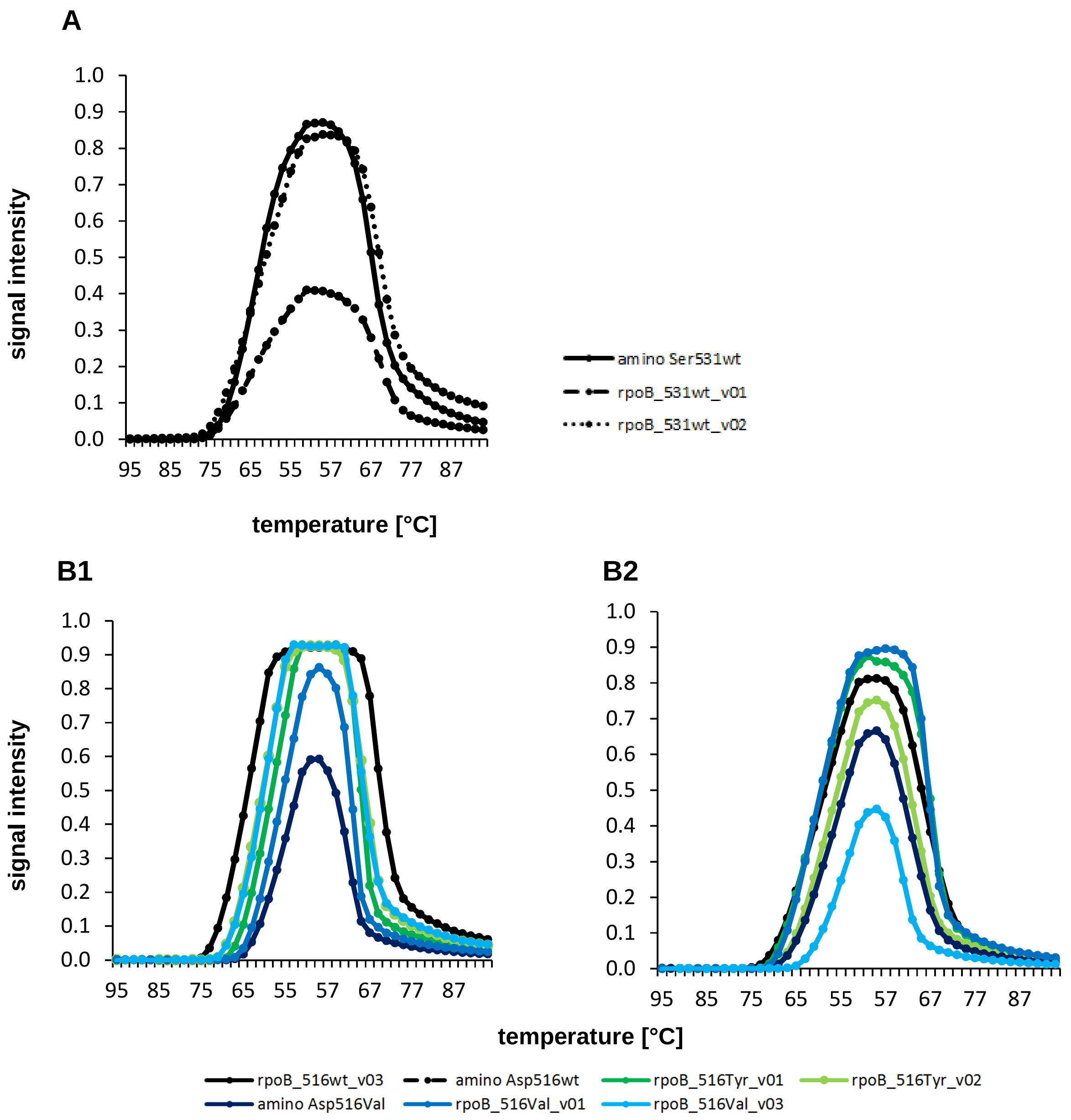

Fig S1. Exemplary melt curve analysis of selected probe and reporter variants. For preselection of reporter oligonucleotide variants a melt curve analysis was done. A: In a first step only reporters with signal intensities > 0.5 were selected (*rpoB* 531 wild type). Therefore the reporter rpoB_531_v01 was excluded due to its low signal intensity of 0.4. B: As second parameter the binding strength of a reporter to its corresponding and non-corresponding probe variants was determined (*rpoB* 516) by the difference in temperature at a defined signal level (0.005). Based on this value the reporter rpoB_516wt_v03 was selected for further experiments (B1) whereas the reporter amino

Asp516wt was excluded (B2) to lower the risk for a weak discrimination between wild type and mutations.
