# Supplementary material for: An application of competitive reporter monitored amplification (CMA) for rapid detection of single nucleotide polymorphisms (SNPs)
Source: PLoS One. 2017 Aug 29;12(8):e0183561. doi: 10.1371/journal.pone.0183561 (PMC5574540; doi:10.1371/journal.pone.0183561)
Supplement: S2 Fig — The multiplex amplification of the targets rpoB, katG, embB and the promotor region of inhA was determined using M. tuberculosis sequence-specific TaqMan probes by Real-Time PCR. Different concentrations of genomic DNA of the M. tuberculosis reference strain H37Rv were tested. The x-axis shows the DNA concentration and the y-axis represents the determined cycle threshold (n = 8). For each target the calibration curve and the calculated efficiency are given which were found to be excellent (E > 0.9). (PDF) [file pone.0183561.s002.pdf]

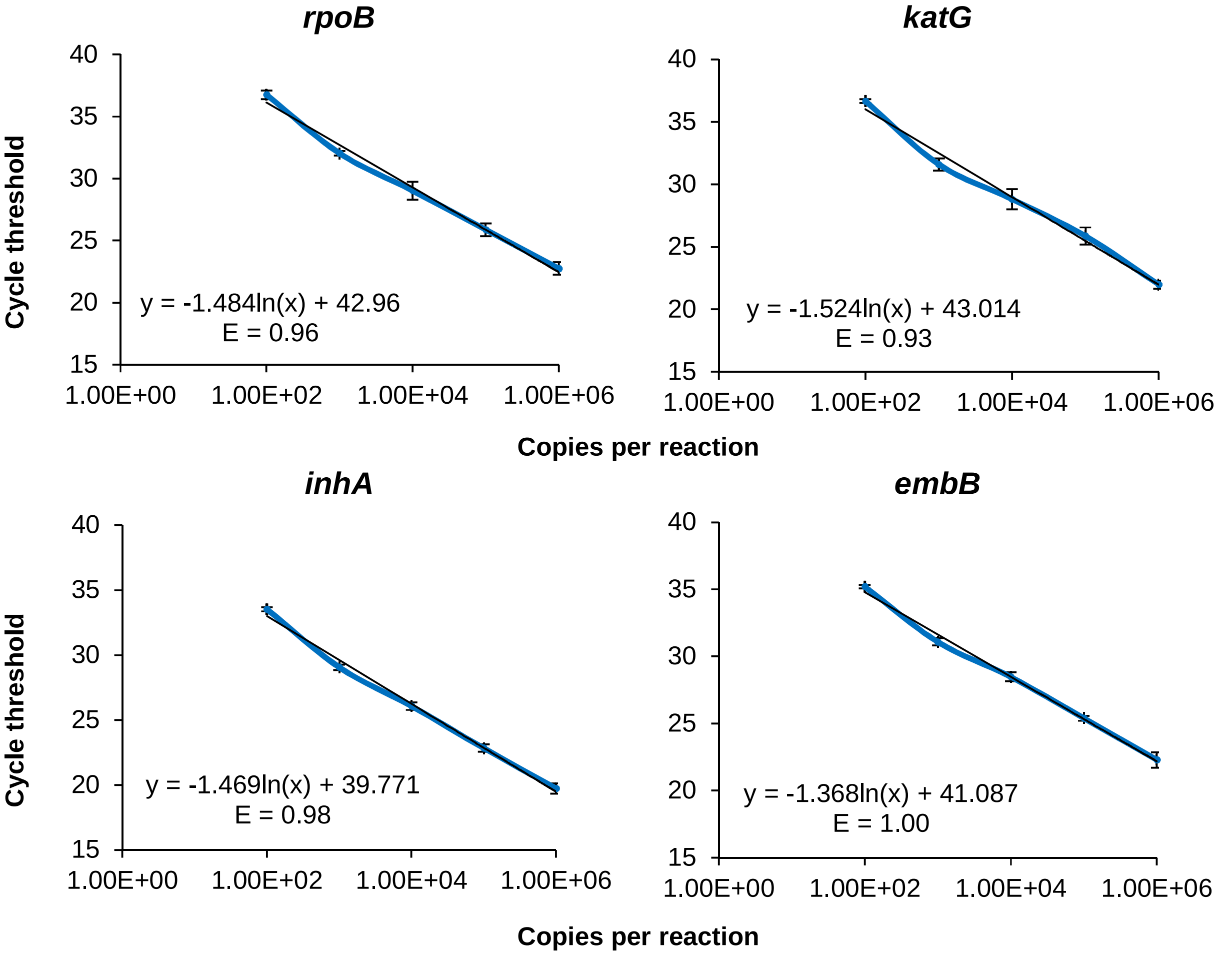

Fig S2. PCR efficiency of the multiplex amplification for all defined *M. tuberculosis* specific targets. The multiplex amplification of the targets *rpoB*, *katG*, *embB* and the promotor region of *inhA* was determined using *M. tuberculosis* sequence-specific TaqMan probes by Real-Time PCR. Different concentrations of genomic DNA of the *M. tuberculosis* reference strain H37Rv were tested. The x-axis shows the DNA concentration and the y-axis represents the determined cycle threshold (n = 8). For each target the calibration curve and the calculated efficiency are given which were found to be excellent (E > 0.9).
