# Supplementary material for: An application of competitive reporter monitored amplification (CMA) for rapid detection of single nucleotide polymorphisms (SNPs)
Source: PLoS One. 2017 Aug 29;12(8):e0183561. doi: 10.1371/journal.pone.0183561 (PMC5574540; doi:10.1371/journal.pone.0183561)
Supplement: S1 Table — (PDF) [file pone.0183561.s003.pdf]

Table S1. Primer used for the preparative PCR and for the CMA-based SNP assay.

Target	Primer	Sequence (5´-3´)	Product size (bp)	Sequence length (bp)	T_m (°C)
rpoB[a]	prep *rpoB* fw	GTCGGCATGTCGCGGATGGAG	327	21	63.3
	prep *rpoB* rv	CGAGCCGATCAGACCGATGTTGG		23	62.8
	rpoB fw	CCGCGATCAAGGAGTTCTTCG	129	21	58.7
	rpoB rv	CACGCTCACGTGACAGACC		19	58.4
katG[b]	prep *katG* fw	GGTCACACTTTCGGTAAGACCCA	268	23	59.6
	prep *katG* rv	CGTCCTTGGCGGTGTATTGC		20	59.3
	katG fw	GCTGGAAGAGCTCGTATGGC	146	20	58.2
	katG rv	AGGGCTCTTCGTCAGCTCC		19	58.7
inhA[c]	prep *inhA* fw	CAAACGTCACGAGCGTAACCC	170	21	59.5
	prep *inhA* rv	AGGACTGAACGGGATACGAATGG		23	59.3
	inhA fw	CGGAAATCGCAGCCACGT	130	18	58.5
	inhA rv	GGACTGAACGGGATACGAATGG		22	58.1
embB[d]	prep *embB* fw	GCTGATTCCGGCAAGCTGG	176	19	59.2
	prep *embB* rv	CGCTGACATGGGTCATCAGC		19	59.1
	embB fw	GACGCCGTGGTGATATTCGG	133	20	58.6
	embB rv	CGAACCAGCGGAAATAGTTGGA		22	58.2

[a] Rv0667; GenBank accession no. NC_000962

[b] Rv1908c; GenBank accession no. NC_000962

[c] Rv1484; GenBank accession no. NC_000962

[d] Rv3795; GenBank accession no. NC_000962
