# Supplementary material for: An application of competitive reporter monitored amplification (CMA) for rapid detection of single nucleotide polymorphisms (SNPs)
Source: PLoS One. 2017 Aug 29;12(8):e0183561. doi: 10.1371/journal.pone.0183561 (PMC5574540; doi:10.1371/journal.pone.0183561)
Supplement: S2 Table — (PDF) [file pone.0183561.s004.pdf]

Table S2. Probe and reporter variants used for the development of the CMA-based SNP assay.

Drug	Locus	Codon	Mutation	Tested probes / reporters	Sequence
Rifampicin	*rpoB*	516	WT	amino Asp516wt	GGGTTGTTCTGGTCCATGAATTGGCTC
				rpoB_516wt_v01	GGGTTGTTCTGGTCCATGAATTGGCTCA
				rpoB_516wt_v03*	AGCGGGTTGTTCTGGTCCATGAATTGG
			Asp516Val	amino Asp516Val	GGGTTGTTCTGGACCATGAATTGGCTC
				rpoB_516Val_v01*	GGGTTGTTCTGGACCATGAATTGGCTCA
				rpoB_516Val_v03	AGCGGGTTGTTCTGGACCATGAATTGG
			Asp516Tyr	rpoB_516Tyr_v01*	CGGGTTGTTCTGGTACATGAATTGGCTCAG
				rpoB_516Tyr_v02	GCGGGTTGTTCTGGTACATGAATTGGCT
		526	WT	amino His526wt*	CGGCGCTTGTGGGTCAACCC
			His526Tyr	amino His526Tyr *	CGGCGCTTGTAGGTCAACCCC
				rpoB_526Tyr_v01	GGCGCTTGTAGGTCAACCCCGA
			His526Asp	amino His526Asp*	TCGGCGCTTGTCGGTCAACC
			His526Arg	rpoB_526Arg_v01	GCGCTTGCGGGTCAACCCC
				rpoB_526Arg_v03*	GTCGGCGCTTGCGGGTCAAC
			His526Asn	rpoB_526Asn_v02	CGGCGCTTGTTGGTCAACCCC
				rpoB_526Asn_v03*	GTCGGCGCTTGTTGGTCAACCC
		531	WT	amino Ser531wt	CCCAGCGCCGACAGTCGG
				rpoB_531wt_v01	CCAGCGCCGACAGTCGGC
				rpoB_531wt_v02	CCCCAGCGCCGACAGTCGG
			Ser531Leu	amino Ser531Leu*	CCCAGCGCCAACAGTCGGC
			Ser531Trp	rpoB_531Trp_v01	CAGCGCCCACAGTCGGCG
				rpoB_531Trp_v03*	GCCCCAGCGCCCACAGTC

Drug	Locus	Codon	Mutation	Tested probes / reporters	Sequence
		533	WT	amino Leu533wt*	GGGCCCCAGCGCCGACA
			Leu533Pro	rpoB_533Pro_v01*	GGCCCCGGCGCCGACA
				rpoB_533Pro_v02	GGCCCCGGCGCCGAC
Isoniazid	*katG*	315	WT	katG_Ser315wt_v01*	CGATGCCGCTGGTGATCGCG
				katG_Ser315wt_v02	CTCGATGCCGCTGGTGATCGC
				katG_Ser315wt_v03	ACCTCGATGCCGCTGGTGATCG
			Ser315 Thr1	katG_Ser315Thr1_v01	CGATGCCGGTGGTGATCGCGT
				katG_Ser315Thr1_v02	CCTCGATGCCGGTGGTGATCGC
				katG_Ser315Thr1_v03*	ACCTCGATGCCGGTGGTGATCG
			Ser315 Thr2	katG_Ser315Thr2_v01	TCGATGCCTGTGGTGATCGCGT
				katG_Ser315Thr2_v02 *	ACCTCGATGCCTGTGGTGATCGC
				katG_Ser315Thr2_v03	CGACCTCGATGCCTGTGGTGATCG
			Ser315Asn	katG_Ser315Asn_v01	CTCGATGCCGTTGGTGATCGCGT
				katG_Ser315Asn_v02 *	ACCTCGATGCCGTTGGTGATCGC
				katG_Ser315Asn_v03	CGACCTCGATGCCGTTGGTGATCG
			Ser315Ile	katG_Ser315Ile_v01	CTCGATGCCGATGGTGATCGCGT
				katG_Ser315Ile_v02	ACCTCGATGCCGATGGTGATCGC
				katG_Ser315Ile_v03 *	CGACCTCGATGCCGATGGTGATCG
	inhA	-15	WT	mabA_up15wt_v01	AACCTATCGTCTCGCCGCGGC
				mabA_up15wt_v02 *	ACAACCTATCGTCTCGCCGCGG
				mabA_up15wt_v03	CCGACAACCTATCGTCTCGCCGC
			15C→T	mabA_up15C>T_v01 *	ACAACCTATCATCTCGCCGCGGC
				mabA_up15C>T_v02	CGACAACCTATCATCTCGCCGCGG
				mabA_up15C>T_v03	CCCGACAACCTATCATCTCGCCGC
		-8	WT	mabA_up08wt_v01 *	CCCCGACAACCTATCGTCTCGCC
				mabA_up08wt_v02	AGTCACCCCGACAACCTATCGTCTCG
				mabA_up08wt_v03	GCAGTCACCCCGACAACCTATCGTCT

Drug	Locus	Codon	Mutation	Tested probes / reporters	Sequence
			8T→A	mabA_up08T>A_v01	ACCCCGACATCCTATCGTCTCGCC
				mabA_up08T>A_v02	AGTCACCCCGACATCCTATCGTCTCG
				mabA_up08T>A_v03 *	GCAGTCACCCCGACATCCTATCGTCT
Ethambutol	*embB*	306	WT	embB_wt_v01	CTCGGGCCATGCCCAGGATGT
				embB_wt_v02	GACTCGGGCCATGCCCAGGA
				embB_wt_v03 *	GCGACTCGGGCCATGCCCA
			Met306Ile1	embB_Met306Ile1_v01	GACTCGGGCTATGCCCAGGATGT
				embB_Met306Ile1_v02 *	CGACTCGGGCTATGCCCAGGATG
				embB_Met306Ile1_v03	GCGACTCGGGCTATGCCCAGG
			Met306Ile2	embB_Met306Ile2_v01 *	GACTCGGGCAATGCCCAGGATGT
				embB_Met306Ile2_v02	CGACTCGGGCAATGCCCAGGA
				embB_Met306Ile2_v03	GCGACTCGGGCAATGCCCAG
			Met306Ile3	embB_Met306Ile3_v01 *	CTCGGGCGATGCCCAGGATGT
				embB_Met306Ile3_v02	GACTCGGGCGATGCCCAGGAT
				embB_Met306Ile3_v03	CGACTCGGGCGATGCCCAGG
			Met306Leu	embB_Met306Leu_v01	CTCGGGCCAGGCCCAGGA
				embB_Met306Leu_v02	ACTCGGGCCAGGCCCAGG
				embB_Met306Leu_v03 *	GACTCGGGCCAGGCCCAGG
			Met306Val	embB_Met306Val_v01	CGGGCCACGCCCAGGATGT
				embB_Met306Val_v02 *	CTCGGGCCACGCCCAGGA
				embB_Met306Val_v03	ACTCGGGCCACGCCCAGG

All oligonucleotides selected for the final array approach are marked with an *.
