# Supplementary material for: An application of competitive reporter monitored amplification (CMA) for rapid detection of single nucleotide polymorphisms (SNPs)
Source: PLoS One. 2017 Aug 29;12(8):e0183561. doi: 10.1371/journal.pone.0183561 (PMC5574540; doi:10.1371/journal.pone.0183561)
Supplement: S3 Table — (PDF) [file pone.0183561.s005.pdf]

Table S3. Target-specific TaqMan probes used for the determination of the Real-Time PCR efficiency.

TaqMan probe	Sequence (5´-3´)	5´ Modification	3´ Modification
rpoB_531wt	CCAGCGCCGACAGTCGG	FAM	BHQ1
katG_315wt	GACCTCGATGCCGCTGGTG	Cy5	BHQ3
inhA_-8wt	CACCCCGACAACCTATCGTCTC	ROX	BHQ2
embB_306wt	CTCGGGCCATGCCCAGGATGT	HEX	BHQ1
