# Supplementary material for: An application of competitive reporter monitored amplification (CMA) for rapid detection of single nucleotide polymorphisms (SNPs)
Source: PLoS One. 2017 Aug 29;12(8):e0183561. doi: 10.1371/journal.pone.0183561 (PMC5574540; doi:10.1371/journal.pone.0183561)
Supplement: S4 Table — The table shows the determined discrimination factors in the upper and the corresponding standard deviations in the lower row, respectively (n = 3 to 4). A discrimination factor < 1 indicates a wild type and a discrimination factor > 1 indicates a mutation. Additionally there is a differentiation between a weak and a strong wild type and mutant detection, respectively. A strong detection is given if the average discrimination factor -/+ 2SD is clearly < 1 for a wild type and > 1 for a mutant detection. If the average discrimination factor -/+ SD is in the range for a wild type or a mutant it is a weak detection. (PDF) [file pone.0183561.s006.pdf]

Table S4. Analysis of wild type genomic DNA and target-specific plasmids carrying different mutations in a multiplex amplification reaction applying all reporter oligonucleotides within the respective target region.

Samples \ Probes	*rpoB* 516Tyr_v01	*rpoB* 516Val_v01	*rpoB* amino526Asp	*rpoB* amino526Tyr	*rpoB* 526Arg_v03	*rpoB* 526Asn_v03	*rpoB* amino531Leu	*rpoB* 531Trp_v03	*rpoB* 533Pro_v01	*katG* 315Asn_v02	*katG* 315Ile_v03	*katG* 315Thr1_v03	*katG* 315Thr2_v02	*inhA* 8T>A_v03	*inhA* 15C>T_v01	*embB* 306Ile1_v02	*embB* 306Ile2_v01	*embB* 306Ile3_v01	*embB* 306Val_v02	*embB* 306Leu_v03
**g_H37Rv**	**0.685**	**0.603**	**0.810**	**0.866**	**0.825**	**0.808**	**0.443**	**0.398**	**0.405**	**0.741**	**0.769**	**0.671**	**0.678**	**0.686**	**0.807**	**0.877**	**0.859**	**0.862**	**0.838**	**0.846**
	0.013	0.044	0.022	0.031	0.027	0.018	0.015	0.014	0.015	0.032	0.018	0.022	0.022	0.003	0.027	0.024	0.032	0.035	0.030	0.048
**p_rpoB516Tyr**	**1.279**	0.828	0.819	0.884	0.830	0.814	0.502	0.460	0.471	---	---	---	---	---	---	---	---	---	---	---
	0.049	0.009	0.022	0.014	0.013	0.046	0.031	0.039	0.039	---	---	---	---	---	---	---	---	---	---	---
**p_rpoB516Val**	1.001	**1.338**	0.792	0.882	0.830	0.777	0.460	0.405	0.415	---	---	---	---	---	---	---	---	---	---	---
	0.022	0.162	0.070	0.054	0.064	0.093	0.118	0.111	0.115	---	---	---	---	---	---	---	---	---	---	---
**p_rpoB526Asp**	0.647	0.556	**1.625**	1.021	0.941	1.050	0.411	0.335	0.344	---	---	---	---	---	---	---	---	---	---	---
	0.027	0.014	0.110	0.113	0.071	0.107	0.026	0.023	0.025	---	---	---	---	---	---	---	---	---	---	---
**p_rpoB526Tyr**	0.575	0.499	0.945	**1.498**	0.914	0.975	0.353	0.302	0.310	---	---	---	---	---	---	---	---	---	---	---
	0.024	0.006	0.023	0.090	0.033	0.027	0.015	0.020	0.017	---	---	---	---	---	---	---	---	---	---	---
**p_rpoB526Arg**	0.614	0.599	0.921	0.881	**1.830**	0.850	0.529	0.456	0.470	---	---	---	---	---	---	---	---	---	---	---
	0.032	0.016	0.011	0.018	0.162	0.031	0.060	0.060	0.056	---	---	---	---	---	---	---	---	---	---	---
**p_rpoB526Asn**	0.668	0.579	0.995	1.010	0.924	**1.164**	0.416	0.355	0.363	---	---	---	---	---	---	---	---	---	---	---
	0.022	0.022	0.016	0.019	0.022	0.048	0.017	0.020	0.018	---	---	---	---	---	---	---	---	---	---	---
**p_rpoB531Leu**	0.644	0.562	0.604	0.810	0.769	0.620	**2.259**	0.986	0.932	---	---	---	---	---	---	---	---	---	---	---
	0.022	0.029	0.017	0.021	0.021	0.038	0.145	0.022	0.016	---	---	---	---	---	---	---	---	---	---	---
**p_rpoB531Trp**	0.745	0.672	0.681	0.816	0.762	0.664	1.156	**1.950**	0.940	---	---	---	---	---	---	---	---	---	---	---
	0.034	0.062	0.076	0.009	0.059	0.040	0.029	0.235	0.008	---	---	---	---	---	---	---	---	---	---	---
**p_rpoB533Pro**	0.641	0.589	0.700	0.874	0.827	0.705	0.819	0.718	**1.812**	---	---	---	---	---	---	---	---	---	---	---
	0.058	0.103	0.132	0.029	0.043	0.076	0.097	0.061	0.335	---	---	---	---	---	---	---	---	---	---	---

Samples \ Probes	*rpoB* 516Tyr_v01	*rpoB* 516Val_v01	*rpoB* amino526Asp	*rpoB* amino526Tyr	*rpoB* 526Arg_v03	*rpoB* 526Asn_v03	*rpoB* amino531Leu	*rpoB* 531Trp_v03	*rpoB* 533Pro_v01	*katG* 315Asn_v02	*katG* 315Ile_v03	*katG* 315Thr1_v03	*katG* 315Thr2_v02	*inhA* 8T>A_v03	*inhA* 15C>T_v01	*embB* 306Ile1_v02	*embB* 306Ile2_v01	*embB* 306Ile3_v01	*embB* 306Val_v02	*embB* 306Leu_v03
**p_katG315Asn**	---	---	---	---	---	---	---	---	---	**1.511**	1.155	0.923	0.877	---	---	---	---	---	---	---
	---	---	---	---	---	---	---	---	---	0.115	0.078	0.044	0.048	---	---	---	---	---	---	---
**p_katG315Ile**	---	---	---	---	---	---	---	---	---	1.022	**1.750**	0.910	0.873	---	---	---	---	---	---	---
	---	---	---	---	---	---	---	---	---	0.014	0.016	0.018	0.009	---	---	---	---	---	---	---
**p_katG315Thr1**	---	---	---	---	---	---	---	---	---	1.019	1.162	**1.213**	1.041	---	---	---	---	---	---	---
	---	---	---	---	---	---	---	---	---	0.077	0.025	0.088	0.059	---	---	---	---	---	---	---
**p_katG315Thr2**	---	---	---	---	---	---	---	---	---	0.916	0.928	0.896	**1.200**	---	---	---	---	---	---	---
	---	---	---	---	---	---	---	---	---	0.046	0.032	0.054	0.057	---	---	---	---	---	---	---
**p_inhA-8T>A**	---	---	---	---	---	---	---	---	---	---	---	---	---	**1.945**	0.807	---	---	---	---	---
	---	---	---	---	---	---	---	---	---	---	---	---	---	0.175	0.021	---	---	---	---	---
**p_inhA-15C>T**	---	---	---	---	---	---	---	---	---	---	---	---	---	0.941	**2.246**	---	---	---	---	---
	---	---	---	---	---	---	---	---	---	---	---	---	---	0.038	0.125	---	---	---	---	---
**p_embB306Ile1**	---	---	---	---	---	---	---	---	---	---	---	---	---	---	---	**1.929**	1.176	1.044	0.993	0.995
	---	---	---	---	---	---	---	---	---	---	---	---	---	---	---	0.214	0.030	0.044	0.030	0.006
**p_embB306Ile2**	---	---	---	---	---	---	---	---	---	---	---	---	---	---	---	1.263	**2.543**	1.130	1.009	1.019
	---	---	---	---	---	---	---	---	---	---	---	---	---	---	---	0.047	0.321	0.023	0.019	0.022
**p_embB306Ile3**	---	---	---	---	---	---	---	---	---	---	---	---	---	---	---	1.364	1.479	**2.457**	0.982	0.991
	---	---	---	---	---	---	---	---	---	---	---	---	---	---	---	0.056	0.272	0.286	0.020	0.017
**p_emB306Val**	---	---	---	---	---	---	---	---	---	---	---	---	---	---	---	0.997	1.001	1.000	**1.349**	1.004
	---	---	---	---	---	---	---	---	---	---	---	---	---	---	---	0.003	0.016	0.008	0.051	0.012
**p_embB306Val**	---	---	---	---	---	---	---	---	---	---	---	---	---	---	---	0.931	0.922	0.930	0.928	**1.536**
	---	---	---	---	---	---	---	---	---	---	---	---	---	---	---	0.014	0.008	0.028	0.012	0.141

strong wild type discrimination
weak wild type discrimination
strong mutant discrimination
weak mutant discrimination
no discrimination possible

The table shows the determined discrimination factors in the upper and the corresponding standard deviations in the lower row, respectively (n = 3 to 4). A discrimination factor < 1 indicates a wild type and a discrimination factor > 1 indicates a mutation. Additionally there is a differentiation between a weak and a strong wild type and mutant detection, respectively. A strong detection is given if the average discrimination factor -/+ 2SD is clearly < 1 for a wild type and > 1 for a mutant detection. If the average discrimination factor -/+ SD is in the range for a wild type or a mutant it is a weak detection.
