# Supplementary material for: An application of competitive reporter monitored amplification (CMA) for rapid detection of single nucleotide polymorphisms (SNPs)
Source: PLoS One. 2017 Aug 29;12(8):e0183561. doi: 10.1371/journal.pone.0183561 (PMC5574540; doi:10.1371/journal.pone.0183561)
Supplement: S5 Table — The table shows the determined discrimination factors as well as the standard deviations (n = 3 to 5) for all analyzed target-specific plasmids. A strong wild type or mutant detection is given if the average discrimination factor -/+ 2SD is <1 or >1, respectively. A weak detection was determined if the average discrimination factor -/+SD was < 1 or > 1, respectively. (PDF) [file pone.0183561.s007.pdf]

Table S5. Analysis of wild type genomic DNA and target-specific plasmids carrying different mutant genotypes in a multiplex amplification reaction applying all reporter oligonucleotides for all defined target regions.

Samples \ Probes	*rpoB* 516Tyr_v01	*rpoB* 516Val_v01	*rpoB* amino526Asp	*rpoB* amino526Tyr	*rpoB* 526Arg_v03	*rpoB* 526Asn_v03	*rpoB* amino531Leu	*rpoB* 531Trp_v03	*rpoB* 533Pro_v01	*katG* 315Asn_v02	*katG* 315Ile_v03	*katG* 315Thr1_v03	*katG* 315Thr2_v02	*inhA* 8T>A_v03	*inhA* 15C>T_v01	*embB* 306Ile1_v02	*embB* 306Ile2_v01	*embB* 306Ile3_v01	*embB* 306Val_v02	*embB* 306Leu_v03
**g_H37Rv**	**0.834**	**0.736**	**0.834**	**0.912**	**0.899**	**0.860**	**0.554**	**0.497**	**0.507**	**0.827**	**0.759**	**0.755**	**0.722**	**0.685**	**0.793**	**0.917**	**0.921**	**0.897**	**0.892**	**0.895**
	0.037	0.030	0.056	0.018	0.016	0.046	0.039	0.056	0.054	0.052	0.053	0.025	0.029	0.006	0.009	0.011	0.006	0.010	0.011	0.012
**p_rpoB526Asp**	0.693	0.613	**1.616**	1.026	0.963	1.098	0.415	0.332	0.349	---	---	---	---	---	---	---	---	---	---	---
	0.049	0.040	0.040	0.017	0.004	0.022	0.016	0.020	0.015	---	---	---	---	---	---	---	---	---	---	---
**p_rpoB531Leu**	0.691	0.601	0.613	0.830	0.773	0.671	**2.239**	1.009	0.918	---	---	---	---	---	---	---	---	---	---	---
	0.026	0.021	0.026	0.008	0.008	0.042	0.180	0.016	0.018	---	---	---	---	---	---	---	---	---	---	---
**p_katG315Ile**	---	---	---	---	---	---	---	---	---	**1.300**	0.899	0.888	0.864	---	---	---	---	---	---	---
	---	---	---	---	---	---	---	---	---	0.116	0.105	0.035	0.039	---	---	---	---	---	---	---
**p_katG315Asn**	---	---	---	---	---	---	---	---	---	1.159	**1.445**	1.022	0.980	---	---	---	---	---	---	---
	---	---	---	---	---	---	---	---	---	0.070	0.181	0.021	0.022	---	---	---	---	---	---	---
**p_katG315Thr1**	---	---	---	---	---	---	---	---	---	1.398	1.193	**1.578**	1.216	---	---	---	---	---	---	---
	---	---	---	---	---	---	---	---	---	0.121	0.153	0.122	0.088	---	---	---	---	---	---	---
**p_katG315Thr2**	---	---	---	---	---	---	---	---	---	1.019	1.022	1.026	**1.316**	---	---	---	---	---	---	---
	---	---	---	---	---	---	---	---	---	0.012	0.064	0.022	0.127	---	---	---	---	---	---	---
**p_inhA-15C>T**	---	---	---	---	---	---	---	---	---	---	---	---	---	0.963	**2.008**	---	---	---	---	---
	---	---	---	---	---	---	---	---	---	---	---	---	---	0.011	0.385	---	---	---	---	---
**p_embB306Ile2**	---	---	---	---	---	---	---	---	---	---	---	---	---	---	---	1.128	**1.652**	1.062	0.999	1.006
	---	---	---	---	---	---	---	---	---	---	---	---	---	---	---	0.042	0.267	0.042	0.001	0.015
**p_embB306Val**	---	---	---	---	---	---	---	---	---	---	---	---	---	---	---	1.003	1.015	1.001	**1.254**	1.021
	---	---	---	---	---	---	---	---	---	---	---	---	---	---	---	0.005	0.010	0.005	0.110	0.021

strong wild type discrimination
weak wild type discrimination
strong mutant discrimination
weak mutant discrimination
no discrimination possible

The table shows the determined discrimination factors as well as the standard deviations (n = 3 to 5) for all analyzed target-specific plasmids. A strong wild type or mutant detection is given if the average discrimination factor -/+ 2SD is <1 or >1, respectively. A weak detection was determined if the average discrimination factor -/+SD was < 1 or > 1, respectively.
