# Supplementary material for: An application of competitive reporter monitored amplification (CMA) for rapid detection of single nucleotide polymorphisms (SNPs)
Source: PLoS One. 2017 Aug 29;12(8):e0183561. doi: 10.1371/journal.pone.0183561 (PMC5574540; doi:10.1371/journal.pone.0183561)
Supplement: S6 Table — The table shows the determined discrimination factors and the standard deviations, respectively (n = 3 to 4) if plasmids carrying different mutations were combined. A strong wild type or mutant detection is given if the average discrimination factor -/+ 2SD is < 1 for a wild type and > 1 for a mutant, respectively, whereas values of < 1 or > 1 for the average discrimination factor -/+SD represents a weak detection. (PDF) [file pone.0183561.s008.pdf]

Table S6. Analysis of different plasmid combinations with the CMA-based SNP assay.

Samples \ Probes	*rpoB* 516Tyr_v01	*rpoB* 516Val_v01	*rpoB* amino526Asp	*rpoB* amino526Tyr	*rpoB* 526Arg_v03	*rpoB* 526Asn_v03	*rpoB* amino531Leu	*rpoB* 531Trp_v03	*rpoB* 533Pro_v01	*katG* 315Asn_v02	*katG* 315Ile_v03	*katG* 315Thr1_v03	*katG* 315Thr2_v02	*inhA* 8T>A_v03	*inhA* 15C>T_v01	*embB* 306Ile1_v02	*embB* 306Ile2_v01	*embB* 306Ile3_v01	*embB* 306Val_v02	*embB* 306Leu_v03
**g_H37Rv**	0.842	0.770	0.860	0.902	0.869	0.863	0.669	0.598	0.616	0.888	0.811	0.819	0.859	0.733	0.853	0.920	0.931	0.913	0.909	0.909
	0.036	0.030	0.007	0.009	0.019	0.027	0.021	0.019	0.025	0.036	0.019	0.039	0.046	0.043	0.027	0.004	0.011	0.020	0.013	0.003
**p_rpoB526Asp + p_embB306Val**	0.877	0.855	**1.159**	0.999	0.971	1.013	0.680	0.584	0.624	---	---	---	---	---	---	1.045	1.050	1.045	**1.159**	1.067
	0.030	0.029	0.003	0.002	0.017	0.002	0.055	0.024	0.022	---	---	---	---	---	---	0.041	0.043	0.045	0.019	0.054
**p_rpoB531Leu + p_katG315Ile**	0.822	0.729	0.776	0.898	0.814	0.810	**1.407**	0.960	0.909	**1.490**	1.018	0.971	0.918	---	---	---	---	---	---	---
	0.020	0.028	0.026	0.031	0.032	0.027	0.120	0.117	0.066	0.041	0.062	0.030	0.008	---	---	---	---	---	---	---
**p_rpoB526Asp + p_katG315Thr2 + p_embB306Val**	0.819	0.721	**1.168**	0.956	0.899	0.929	0.596	0.534	0.567	1.002	1.000	1.035	**1.358**	---	---	1.051	1.025	1.018	**1.180**	0.984
	0.015	0.025	0.032	0.030	0.031	0.079	0.033	0.044	0.040	0.021	0.066	0.086	0.093	---	---	0.013	0.016	0.014	0.032	0.006
**p_rpoB531Leu + p_katG315Ile + p_embB306Ile2**	0.816	0.728	0.776	0.899	0.802	0.812	**1.485**	0.960	0.927	**1.449**	1.029	0.990	0.910	---	---	1.187	**1.788**	1.166	0.970	0.975
	0.040	0.041	0.019	0.020	0.017	0.015	0.148	0.050	0.047	0.035	0.013	0.025	0.017	---	---	0.029	0.081	0.033	0.012	0.008

strong wild type discrimination
weak wild type discrimination
strong mutant discrimination
weak mutant discrimination
no discrimination possible

The table shows the determined discrimination factors and the standard deviations, respectively (n = 3 to 4) if plasmids carrying different mutations were combined. A strong wild type or mutant detection is given if the average discrimination factor -/+ 2SD is < 1 for a wild type and > 1 for a mutant, respectively, whereas values of < 1 or > 1 for the average discrimination factor -/+SD represents a weak detection.
