# Supplementary material for: An application of competitive reporter monitored amplification (CMA) for rapid detection of single nucleotide polymorphisms (SNPs)
Source: PLoS One. 2017 Aug 29;12(8):e0183561. doi: 10.1371/journal.pone.0183561 (PMC5574540; doi:10.1371/journal.pone.0183561)
Supplement: S7 Table — The results of tested genomic DNAs (g) and inactivated cell culture material (c) derived from the same strain are listed in the table (n = 3 to 4). The discrimination factors are given in the upper and the standard deviations in the row below, respectively. A wild type is detected if the discrimination factor is < 1. A differentiation is applied between a strong (average discrimination factor + 2SD) and a weak (average discrimination factor + SD) wild type detection. A strong mutant detection is given if the average discrimination factor − 2SD is clear > 1 and a weak mutant detection for an average discrimination factor—SD is > 1. (PDF) [file pone.0183561.s009.pdf]

Table S7. Analysis of genomic DNA and inactivated cell culture material from different *M. tuberculosis* strains.

Probes / Samples	*rpoB* 516Tyr_v01	*rpoB* 516Val_v01	*rpoB* amino526Asp	*rpoB* amino526Tyr	*rpoB* 526Arg_v03	*rpoB* 526Asn_v03	*rpoB* amino531Leu	*rpoB* 531Trp_v03	*rpoB* 533Pro_v01	*katG* 315Asn_v02	*katG* 315Ile_v03	*katG* 315Thr1_v03	*katG* 315Thr2_v02	*inhA* 8T>A_v03	*inhA* 15C>T_v01	*embB* 306Ile1_v02	*embB* 306Ile2_v01	*embB* 306Ile3_v01	*embB* 306Val_v02	*embB* 306Leu_v03
**gH37Rv**	0.682	0.571	0.761	0.828	0.731	0.745	0.410	0.370	0.385	0.752	0.702	0.668	0.667	0.752	0.745	0.847	0.853	0.819	0.808	0.791
	0.022	0.017	0.015	0.053	0.012	0.069	0.013	0.011	0.011	0.010	0.015	0.074	0.013	0.024	0.027	0.013	0.007	0.010	0.009	0.007
**cH37Rv**	0.696	0.624	0.835	0.899	0.851	0.849	0.409	0.372	0.382	0.816	0.752	0.719	0.734	0.778	0.767	0.891	0.876	0.869	0.867	0.863
	0.016	0.019	0.011	0.018	0.013	0.033	0.018	0.017	0.014	0.045	0.067	0.048	0.025	0.026	0.015	0.012	0.009	0.019	0.016	0.014
**g1429/02**	0.718	0.660	0.800	0.887	0.820	0.834	0.458	0.383	0.407	1.080	1.106	1.138	**1.682**	0.676	0.821	0.933	0.914	0.880	0.894	0.889
	0.033	0.042	0.022	0.018	0.014	0.015	0.056	0.050	0.049	0.020	0.023	0.010	0.143	0.013	0.043	0.006	0.013	0.027	0.023	0.034
**c1429/02**	0.663	0.610	0.786	0.879	0.799	0.822	0.405	0.333	0.357	1.092	1.134	1.179	**1.798**	0.664	0.795	0.935	0.881	0.855	0.870	0.854
	0.028	0.022	0.012	0.015	0.012	0.012	0.030	0.023	0.027	0.020	0.021	0.035	0.030	0.014	0.016	0.004	0.007	0.008	0.027	0.008
**g368/01**	0.766	0.709	0.695	0.895	0.779	0.787	**1.866**	0.975	0.887	1.222	1.224	**1.430**	1.207	0.701	0.840	0.909	0.908	0.897	0.885	0.878
	0.099	0.118	0.071	0.029	0.048	0.052	0.324	0.026	0.020	0.093	0.055	0.143	0.030	0.035	0.057	0.022	0.019	0.030	0.034	0.041
**c368/01**	0.687	0.609	0.683	0.877	0.776	0.753	**1.807**	1.008	0.989	1.391	1.269	**1.526**	1.231	0.714	0.695	0.929	0.905	0.904	0.901	0.898
	0.049	0.042	0.023	0.019	0.020	0.012	0.041	0.010	0.016	0.047	0.036	0.083	0.035	0.022	0.012	0.008	0.010	0.010	0.011	0.012
**g4724/03**	0.660	0.550	0.949	0.900	**1.631**	0.887	0.368	0.331	0.347	1.339	1.216	**1.516**	1.149	0.691	0.674	0.896	0.890	0.878	0.840	0.802
	0.022	0.014	0.008	0.010	0.005	0.062	0.003	0.003	0.005	0.008	0.016	0.046	0.009	0.016	0.010	0.026	0.030	0.030	0.028	0.029
**c4724/03**	0.696	0.573	0.925	0.896	**1.646**	0.890	0.368	0.324	0.350	1.454	1.307	**1.542**	1.277	0.728	0.697	0.931	0.897	0.884	0.860	0.812
	0.028	0.016	0.013	0.008	0.034	0.049	0.006	0.003	0.006	0.030	0.022	0.072	0.031	0.001	0.019	0.008	0.016	0.009	0.035	0.017
**g1049/02**	0.746	0.711	0.727	0.912	0.780	0.804	**1.783**	0.962	0.881	1.227	1.239	**1.396**	1.173	0.667	0.835	**1.239**	1.054	1.016	0.984	1.023
	0.090	0.080	0.032	0.018	0.032	0.030	0.145	0.014	0.025	0.100	0.044	0.088	0.028	0.059	0.020	0.026	0.042	0.019	0.008	0.019
**c1049/02**	0.644	0.524	0.604	0.816	0.710	0.629	**2.171**	0.963	0.902	1.550	1.311	**1.676**	1.300	0.723	0.743	**1.933**	1.268	1.162	0.968	0.967
	0.049	0.040	0.024	0.019	0.017	0.034	0.032	0.018	0.003	0.016	0.016	0.055	0.079	0.014	0.014	0.022	0.035	0.014	0.014	0.013

Samples \ Probes		*rpoB* 516Tyr_v01	*rpoB* 516Val_v01	*rpoB* amino526Asp	*rpoB* amino526Tyr	*rpoB* 526Arg_v03	*rpoB* 526Asn_v03	*rpoB* amino531Leu	*rpoB* 531Trp_v03	*rpoB* 533Pro_v01	*katG* 315Asn_v02	*katG* 315Ile_v03	*katG* 315Thr1_v03	*katG* 315Thr2_v02	*inhA* 8T>A_v03	*inhA* 15C>T_v01	*embB* 306Ile1_v02	*embB* 306Ile2_v01	*embB* 306Ile3_v01	*embB* 306Val_v02	*embB* 306Leu_v03
**g2822/06**		0.705	0.614	**1.606**	1.004	0.918	1.043	0.405	0.293	0.298	0.823	0.742	0.674	0.706	1.004	**1.847**	1.009	1.005	1.008	**1.196**	0.996
		0.028	0.030	0.043	0.018	0.016	0.067	0.015	0.012	0.016	0.011	0.088	0.018	0.050	0.007	0.039	0.004	0.002	0.004	0.020	0.003
**c2822/06**		0.694	0.604	**1.389**	0.994	0.985	1.058	0.387	0.355	0.353	0.800	0.721	0.681	0.695	1.004	**1.767**	1.003	0.998	0.997	**1.174**	0.994
		0.013	0.012	0.024	0.015	0.017	0.032	0.020	0.018	0.016	0.006	0.051	0.008	0.011	0.003	0.113	0.003	0.004	0.004	0.019	0.007
**g9975/05**		0.692	0.587	0.646	0.859	0.739	0.720	**2.164**	0.974	0.912	1.348	1.260	**1.482**	1.212	0.744	0.736	1.014	1.042	1.036	**1.199**	0.986
		0.036	0.043	0.051	0.036	0.047	0.038	0.284	0.026	0.009	0.009	0.037	0.075	0.068	0.070	0.014	0.041	0.032	0.036	0.060	0.015
**c9975/05**		0.725	0.651	0.700	0.894	0.771	0.757	**1.753**	1.008	0.995	1.343	1.236	**1.480**	1.212	0.693	0.702	0.978	0.976	0.976	**1.138**	0.971
		0.004	0.005	0.011	0.012	0.017	0.023	0.052	0.004	0.003	0.012	0.027	0.048	0.006	0.016	0.023	0.041	0.039	0.044	0.053	0.039

strong wild type discrimination
weak wild type discrimination
strong mutant discrimination
weak mutant discrimination
no discrimination possible

The results of tested genomic DNAs (g) and inactivated cell culture material (c) derived from the same strain are listed in the table (n = 3 to 4). The discrimination factors are given in the upper and the standard deviations in the row below, respectively. A wild type is detected if the discrimination factor is < 1. A differentiation is applied between a strong (average discrimination factor + 2SD) and a weak (average discrimination factor + SD) wild type detection. A strong mutant detection is given if the average discrimination factor - 2SD is clear > 1 and a weak mutant detection for an average discrimination factor - SD is > 1.
